# Ha-Ras stabilization mediates pro-fibrotic signals in dermal fibroblasts

**DOI:** 10.1186/1755-1536-4-8

**Published:** 2011-03-01

**Authors:** Silvia Smaldone, Jacopo Olivieri, Gabriele Luca Gusella, Gianluca Moroncini, Armando Gabrielli, Francesco Ramirez

**Affiliations:** 1 Mount Sinai School of Medicine, Department of Pharmacology and Systems Therapeutics, One Gustave L Levy Place, Box 1603, New York, NY 10029, USA; 2 University of Ancona, Istituto di Patologia Medica e Metodolgia Clinica, Piazza Roma 22, 60121 Ancona, Italy; 3 Mount Sinai School of Medicine, Department of Medicine and Nephrology, One Gustave L Levy Place, New York, NY 10029, USA

## Abstract

**Background:**

Scleroderma (systemic sclerosis; SSc) is a clinically heterogeneous and often lethal acquired disorder of the connective tissue that is characterized by vascular, immune/inflammatory and fibrotic manifestations. Tissue fibrosis is the main cause of morbidity and mortality in SSc and an unmet medical challenge, mostly because of our limited understanding of the molecular factors and signalling events that trigger and sustain disease progression. Recent evidence has correlated skin fibrosis in SSc with stabilization of proto-oncogene Ha-Ras secondary to auto-antibody stimulation of reactive oxygen species production. The goal of the present study was to explore the molecular connection between Ha-Ras stabilization and collagen I production, the main read-out of fibrogenesis, in a primary dermal fibroblast culture system that replicates the early stages of disease progression in SSc.

**Results:**

Forced expression of proto-oncogene Ha-Ras in dermal fibroblasts demonstrated the promotion of an immediate collagen I up-regulation, as evidenced by enhanced activity of a collagen I-driven luciferase reporter plasmid and increased accumulation of endogenous collagen I proteins. Moreover, normal levels of *Tgfβ *transcripts and active transforming growth factor-beta (TGFβ) implied Ha-Ras stimulation of the canonical Smad2/3 signalling pathway independently of TGFβ production or activation. Heightened Smad2/3 signalling was furthermore correlated with greater Smad3 phosphorylation and Smad3 protein accumulation, suggesting that Ha-Ras may target both Smad2/3 activation and turnover. Additional *in vitro *evidence excluded a contribution of ERK1/2 signalling to improper Smad3 activity and collagen I production in cells that constitutively express Ha-Ras.

**Conclusions:**

Our study shows for the first time that constitutively elevated Ha-Ras protein levels can directly stimulate Smad2/3 signalling and collagen I accumulation independently of TGFβ neo-synthesis and activation. This finding therefore implicates the Ha-Ras pathway with the early onset of fibrosis in SSc and implicitly identifies new therapeutic targets in SSc.

## Background

Wound healing is a complex and tightly regulated physiological process that involves several different cell types and a plethora of signalling molecules [1-3]. In the early phase of this process, platelets brought by the blood stream form a fibrin cloth at the site of injury that blocks bleeding (haemostasis). Increased levels of soluble signals, induced by the cell-mediated inflammatory response, subsequently promote migration and proliferation of angiogenic cells and activated fibroblasts (myofibroblasts) that synthesize extracellular matrix (ECM) proteins, chiefly collagen I [1]. By contracting the newly synthesized ECM, myofibroblasts allow the closure of the wound where the provisional matrix is ultimately remodelled to form a scar [1]. Failure of myofibroblasts to terminate the wound healing process results in excessive accumulation and contraction of a poorly organized ECM. Unopposed myofibroblasts activation in fibrotic conditions, such as scleroderma (SSc), causes gradual and irreversible alteration of connective tissue architecture with deleterious consequences for organ function. In spite of significant investigative effort, our current knowledge of the molecular and cellular events that promote and sustain myofibroblasts activation is limited and consequently, the clinical management of affected patients remains confined to therapies that alleviate secondary symptoms rather than arresting the often fatal consequences of the fibrotic response.

Clinical findings, cell culture experiments and animal models have firmly established the prominent role that transforming growth factor-β (TGFβ) plays in modulating the physiological process of wound healing and in driving the pathological sequence of fibrotic responses [2,3]. Even though genetic or pharmacological interference of TGFβ signalling in rodents can mitigate fibrotic disease, they can also result in severe side effects due to the wide range of biological processes that involve this multifunctional cytokine [2]. It follows that a better understanding of molecular events upstream, downstream or parallel to improper TGFβ signalling represents a pre-requisite to the development of more effective and safer therapies for fibrotic conditions.

TGFβ signals through the activation of a membrane-receptor serine/threonine kinase complex that phosphorylates the Smad2 and Smad3 proteins [receptor-activated Smads (R-Smad); canonical TGFβ signalling pathway] [4]. Activated R-Smad proteins associate with Smad4 to migrate into the nucleus and modulate the expression of several different genes together with transcriptional co-activators and co-repressors [4]. In addition to the canonical R-Smad pathway, TGFβ can also stimulate the activity of mitogen-activated protein kinases (MAPKs; non-canonical TGFβ signalling pathway) and MAPKs and other stress response pathways can, in turn, modulate R-Smad signalling with discrete intracellular outcomes [5]. For example, the proto-oncogene Ha-Ras can stimulate or inhibit R-Smad signalling, operate upstream of TGFβ by promoting its auto-induction or act independently of the canonical TGFβ signalling pathway [6-10]. Hence, complex interactions amongst different signalling pathways are believed to specify contextual responses of the cells to diverse environmental stimuli.

The Ras gene family comprises three genetically distinct but structurally related proteins (Ha-Ras, Ki-Ras and N-Ras), which operate as molecular switches that cycle between an inactive GDP (guanosine diphosphate)-bound to an active GTP (guanosine triphosphate)-bound form [11]. Ras family members have functionally distinct roles that are dictated by their intracellular localization and the cellular context [11]. Ras signalling is the nodal point of multiple extracellular cues, including the profibrotic signals of TGFβ, angiotensin II, platelet-derived growth factor (PDGF) and reactive oxygen species (ROS) [7,12-14]. Recent studies of SSc cells have causally connected circulating auto-antibodies against PDGF receptors (PDGFR) with the stimulation of ROS production, Ha-Ras stabilization and collagen I overproduction [15,16]. However, the contribution of Ha-Ras activity to SSc fibrogenesis, as well as the cross-talk between Ha-Ras and TGFβ signalling in this disease process remains to be fully explored.

It was the scope of the present study to investigate the pro-fibrotic potential of Ha-Ras signalling by using a cell culture system that replicates the downstream events previously described in SSc myofibroblasts. Our results show for the first time that constitutively elevated Ha-Ras protein levels promote R-Smad signalling and collagen I accumulation independently of TGFβ synthesis and activation. These findings implicitly connect the Ha-Ras pathway with the onset of fibrosis through the stimulation of canonical TGFβ pathway, even though the biochemical identity of the connection was not investigated here. More generally, our work provides new insight into early disease-causing events that could in principle represent new therapeutic targets in fibrotic conditions like scleroderma.

## Materials and methods

### Cell cultures

Human fetal dermal fibroblasts (hDF; GM06111) were purchased from the Human Genetic Mutant Cell Repository (NJ, USA). Cells were maintained at 37°C in a sterile and humidified atmosphere of 5% CO_2_. Cells were grown in Dulbecco's modified Eagle's medium (DMEM) containing 10% fetal bovine serum (FBS; Atlanta Biologicals, GA, USA) and supplemented with streptomycin, penicillin and fungizone. Primary mouse dermal fibroblast (mDF) cultures were established from the dorsal skin of 4-day-old wild-type mice and grown as described above. Several 8 mm sterile skin punches were made from each newborn mice, freed of the subcutaneous tissue by scraping, and laid flat individually into a 10-cm^2 ^tissue culture plate with the dermal side down. Explants were incubated at 37°C for 5 min to let skin adhere. Ten millilitres of medium was added into each plate and cells were allowed to migrate out of the explants for 10 days. Once confluent, cells were trypsinized and either stored in liquid nitrogen or employed immediately: in both cases, cells between passages 1 and 3 were used.

### Cell transfections

Primary mDF and hDF were seeded the day before transfection at a density of 10,000 cells/cm^2 ^and cultured in 0.2% FBS. Cells were transiently co-transfected using Lipofectamine 2000 (Invitrogen, CA, USA) with 0.5 ng of the control plasmid SV40:Renilla-Luc (Promega, WI, USA) and 200 ng of the COL1A2 (human pro-α2 (I) collagen gene) reporter plasmid containing wild-type or mutant TbRE (TGFβ-responsive element) sites [17] or the Smad3 responsive plasmid (CAGA)_12_MLP-Luc (a kind gift of Dr Joan Massagué). In some experiments, the COL1A2 reporter was transiently co-transfected with plasmids expressing wild-type or constitutively active (*V12 *variant) proto-oncogene Ha-Ras (Ha-Ras/pSG5 and ca-Ras/pSG5, respectively) or dominant-negative (N17 variant) Ha-ras (DN-Ras/pSG5; kindly provided by Dr Enrico Avvedimento), or with a plasmid expressing dominant-negative (MH2 deletion) Smad3 (DN-Smad3) [17]. In other transfection experiments, hDF cultures were treated with 20 μg/mL neutralizing pan-TGFβ antibody (MAB1835, R&D System, MN, USA). Luciferase assays were performed 16 h and 24 h after cell transfection and the results were evaluated as previously described [17]. Statistical analyses were performed for all of the experiments using Student's *t *test, assuming a *P *value of ≤ 0.05 as significant.

### Lentiviral infections

A lentivirus expressing the wild-type proto-oncogene Ha-Ras was generated by mutating Ha-Ras *V12 *coding sequence using the quick change II site direct mutagenesis kit (Stratagene, CA, USA) following the manufacturer's instructions. Ha-Ras coding sequence was subcloned into the VVCW/BE lentiviral expression plasmid after EcoR1/EcoRV double digestion. Ha-Ras/VVCW/BE or VVCW/BE empty vector were cotransfected with CMVδR8.2 and pMD.G vectors into 293T packaging cell line as described previously [18]. Viral supernatants were collected 48 h and 72 h after transfection and used to infect mDF cells in the presence of 10 μg/mL of polybrene.

### Immunoblots and immunocyotochemistry

mDF cultured for 2 days in 0.2% FBS were infected with Ha-Ras expressing and control lenti-particles for the indicated length of time. In some experiments, the culture medium included neutralizing pan-TGFβ antibody or MEK inhibitor (PD98059) at concentrations of 10 μM (Calbiochem-EMD Biosciences, NJ, USA). Cell layers were scraped into ice-cold Tris-buffered saline solution (pH 7.4) and flash frozen in liquid nitrogen. Cell extracts were prepared and assayed for total protein content using the BCA kit (Pierce, IL, USA). Protein extracts (10-25 μg/lane) were fractioned by 10% or 6% (w/v) SDS-PAGE and electroblotted onto an Immobilon-P membrane (Millipore, MA, USA). Membranes were incubated first with antibodies against *p*-Smad3 (Invitrogen) or Smad3 (Zymed; 1:1000 dilution) and subsequently with HRP-conjugated anti-rabbit IgG antibody (1:25,000 dilution; Jackson ImmunoResearch Laboratories, PA, USA). Immunoreactive products were visualized by chemiluminescence using the ECL Plus kit (Amersham Biosciences, Amersham, UK) and their relative intensity was evaluated with the aid of Adobe Photoshop software (Adobe Systems Inc, CA, USA). Actin myofibres were visualized in cells infected with Ha-Ras and control lentivirus using antibodies against α-smooth muscle actin (αSMA; Chemicon, CA, USA).

### RNA analyses

For real-time quantitative PCR (qPCR), total RNA was isolated by RNeasy Mini kit (Qiagen, Hilden, Germany) from mDF and used as template for complimentary DNA (cDNA) synthesis (AffinityScript multiple temperature reverse transcriptase, Stratagene). Target complimentary DNAs were amplified using SYBR Green Supermix with ROX (6-carboxy-X-rhodamine; Fermentas) on a Mastercycler ep Realplex instrument (Eppendorf, Hamburg, Germany) at the following thermal cycling conditions: 95°C for 10 min followed by 40 cycles consisting of 95°C for 15 s denaturation, 60°C for 30 s annealing and 72°C for 30 s extension. Amplification primer sets were purchased from SuperArray Bioscience Corporation (Maryland, USA. Comparative quantification was carried out using multiple replicates that were analysed in triplicate. Statistical significance was evaluated by an unpaired *t *test assuming significant association at *P *< 0.05 compared with control samples.

### TGFβ bioassays

Cells for TGFβ bioassays (a kind gift of Dr Daniel Rifkin [19]) were seeded at 60,000 cells/cm^2 ^and then incubated with conditioned media collected from mDF, 6 h and 24 h after lentiviral infection. TGFβ activity was assessed 16 h later in cell lysates by measuring luciferase activity with a TD-20 luminometer (Turner Designs, CA, USA) as described previously [19]. Bioassays were performed in triplicate with multiple samples and evaluated using an unpaired *t *test (MS Excel); significant association was defined when *P *< 0.05 compared with control.

## Results

### Proto-oncogene Ha-Ras increases COL1A2 expression through R-Smad activation

**Figure 1:**
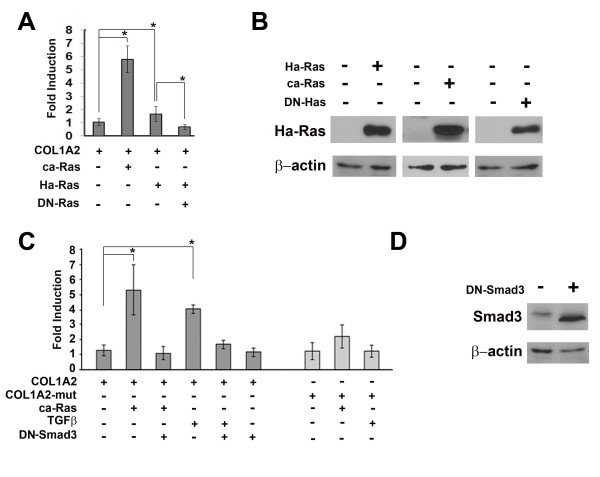
**Smad3-dependent Ha-Ras activation of COL1A2 (human pro-α2 (I) collagen gene)**. (A) Luciferase activity (expressed as fold induction over control sample) of the COL1A2 promoter transiently co-transfected with plasmids expressing Ha-Ras, ca-Ras or DN-Ras in quiescent (human dermal fibroblast) hDF cultures (B) Western blot analyses of recombinant Ha-Ras, ca-Ras and DN-Ras levels in hDF cells (*n *= 3 per each sample) using anti-Ha-Ras antibodies. (C) Luciferase activity (expressed as fold induction over control sample) of wild-type and TbRE [transforming growth factor-β (TGFβ)-responsive element] mutated COL1A2 promoter constructs transiently co-transfected in quiescent hDF cultures with the indicated combinations of ca-Ras expressing plasmid or the DN-Smad3 expression vector; cells stimulated with recombinant TGFβ1 (2 ng/mL) served as a positive control. (D) Western blot analysis of recombinant DN-Smad3 levels in hDF cells (*n *= 3 per each sample) using anti-Smad3 antibodies. Luciferase values in panels A and C represent the average of three independent transfections each performed in duplicate. Error bars signify ± standard deviation and asterisks indicate statistically significant differences (*P *< 0.05).

The first set of experiments was designed to test the hypothesis that wild-type proto-oncogene Ha-Ras (hereafter referred solely as Ha-Ras) is directly involved in collagen I stimulation and to investigate the underlying mechanism. To this end, primary fetal hDFs were transiently co-transfected with a vector expressing constitutively active Ha-Ras (ca-Ras) and a luciferase reporter plasmid driven by the COL1A2 proximal promoter. The results demonstrated that ca-Ras over-expression results in a ~sixfold increase of COL1A2 promoter activity (Figure 1A). Ha-Ras over-expression similarly resulted in increased COL1A2 promoter activity but to a lesser extent than ca-Ras, perhaps reflecting the inherent activation of the latter compared to the former protein (Figure 1A). Reduced COL1A2 up-regulation in hDF co-expressing Ha-Ras and dominant-negative Ha-Ras (DN-Ras) demonstrated the specificity of the former protein action (Figure 1A). Moreover, comparable amounts of the various Ha-Ras versions excluded the formal possibility that the activity of individual expression constructs might account for the observed changes in the transcription from the COL1A2 promoter (Figure 1B).

The COL1A2 proximal promoter contains a TGFβ responsive element (TbRE) that mediates transcriptional up-regulation through the binding of a multiprotein complex that includes R-Smads, Sp1 and p300/CBP and that is also targeted by other pro-fibrotic stimuli, such as those trigged by acetaldehyde, sphingolipids and oncostatin M. [3]. Accordingly, we assessed the potential involvement of R-Smad pathway and the TbRE in Ha-Ras-mediated collagen up-regulation. Two lines of evidence strongly suggested that Ha-Ras stimulates COL1A2 expression, in part, through the binding of activated R-Smad complexes to the TbRE. First, mutations in the Smad3-binding site of the TbRE significantly reduced ca-Ras up-regulation of the COL1A2 promoter (Figure 1C); second, co-transfection of a DN-Smad3 expression plasmid abrogated ca-Ras ability to increase COL1A2 promoter activity (Figure 1C). Once again, immunoblots documented appreciable levels of recombinant DN-Smad3 (Figure 1D).

**Figure 2:**
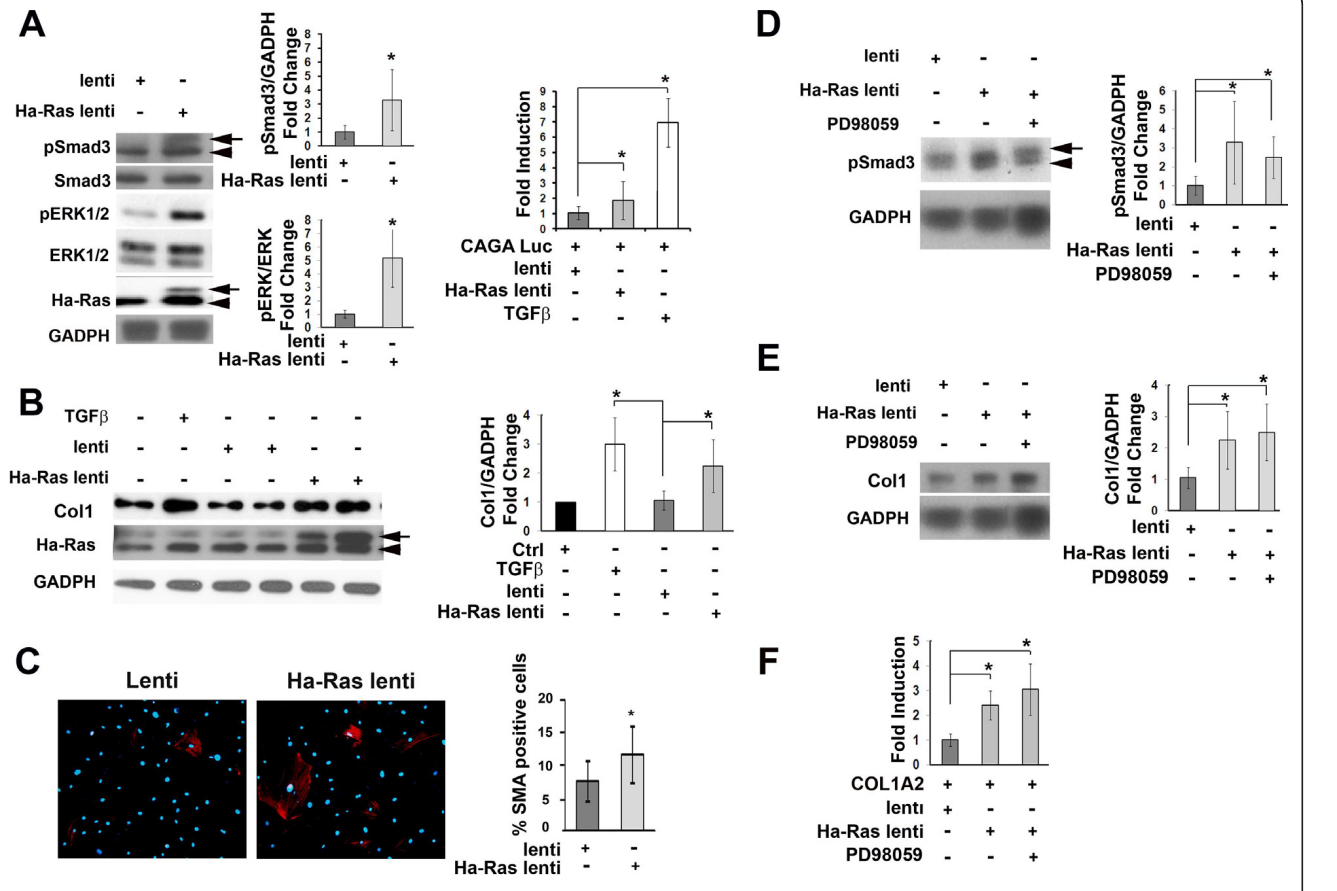
**Activated phenotype of mouse dermal fibroblast (mDF) constitutively expressing Ha-Ras**. (A) Left panel, pSmad3, Smad3, pERK1/2, ERK1/2, Ha-Ras and glyceraldehyde 3-phosphate dehydrogenase (GAPDH) immunoblots of protein extracts from mDF cultures infected with Ha-Ras (Ha-Ras lenti) or control (lenti) lentiviruses at 6 h post-infection (*n *= 3 per each sample) with the bar graphs on the side summarizing the relative ratios of pSmad3 over GADPH and pERK1/2 over ERK1/2; right panel, luciferase activity of a Smad3-responsive plasmid transiently transfected in mDF infected with Ha-Ras or control lentivirus and/or stimulated with recombinant transforming growth factor-β (TGFβ1; 2 ng/mL) for 6 h. (B) Collagen I and Ha-Ras immunoblots of protein extracts from mDF cultures infected with Ha-Ras or control lentiviruses at 24 h post-infection and in control cells treated with recombinant TGFβ1 (2 ng/mL) for the same length of time (*n *= 3 per each sample). (C) α-smooth muscle actin (αSMA) immunostaining of mDF infected with Ha-Ras or control lentiviruses with bar graphs on the side summarizing the percentage of αSMA-positive cells 24 h after the infection; measurements were performed on >100 cells from three independent infections. (D) pSmad3 immunoblots of protein extracts from mDF cultures treated with the MEK inhibitor PD98059 (10 μM) for 2 h prior to infection for 6 h with Ha-Ras or control lentiviruses with bar graphs on the side summarizing the relative ratio of pSmad3 and the loading control GADPH in the various experimental samples (*n *= 3 per each sample). (E) Collagen I immunoblots of protein extracts from mDF cultures treated with the MEK inhibitor PD98059 (10 μM) for 2 h prior to infection with Ha-Ras or control lentiviruses (*n *= 3 per each sample); protein levels were assessed 24 h post-infection and the bar graphs on the side summarize the relative ratio of collagen I and the loading control GADPH in the various experimental samples. (F) Luciferase activity (expressed as fold induction over control sample) of the COL1A2 promoter plasmid transiently transfected in primary mDF infected with Ha-Ras or control lentiviruses at 24 h post-infection with or without 2 h pre-treatment with the MEK inhibitor PD98059 (10 μM). In the relevant panels, arrows and arrowheads point to Ha-Ras and a non-specific immunoreactive products or pSmad3 and an unidentified cross-reacting R-Smad, respectively; luciferase values represent the average of three independent transfections each performed in duplicate; error bars signify ± standard deviation and asterisks indicate statistically significant differences (*P *< 0.05).

**Figure 3:**
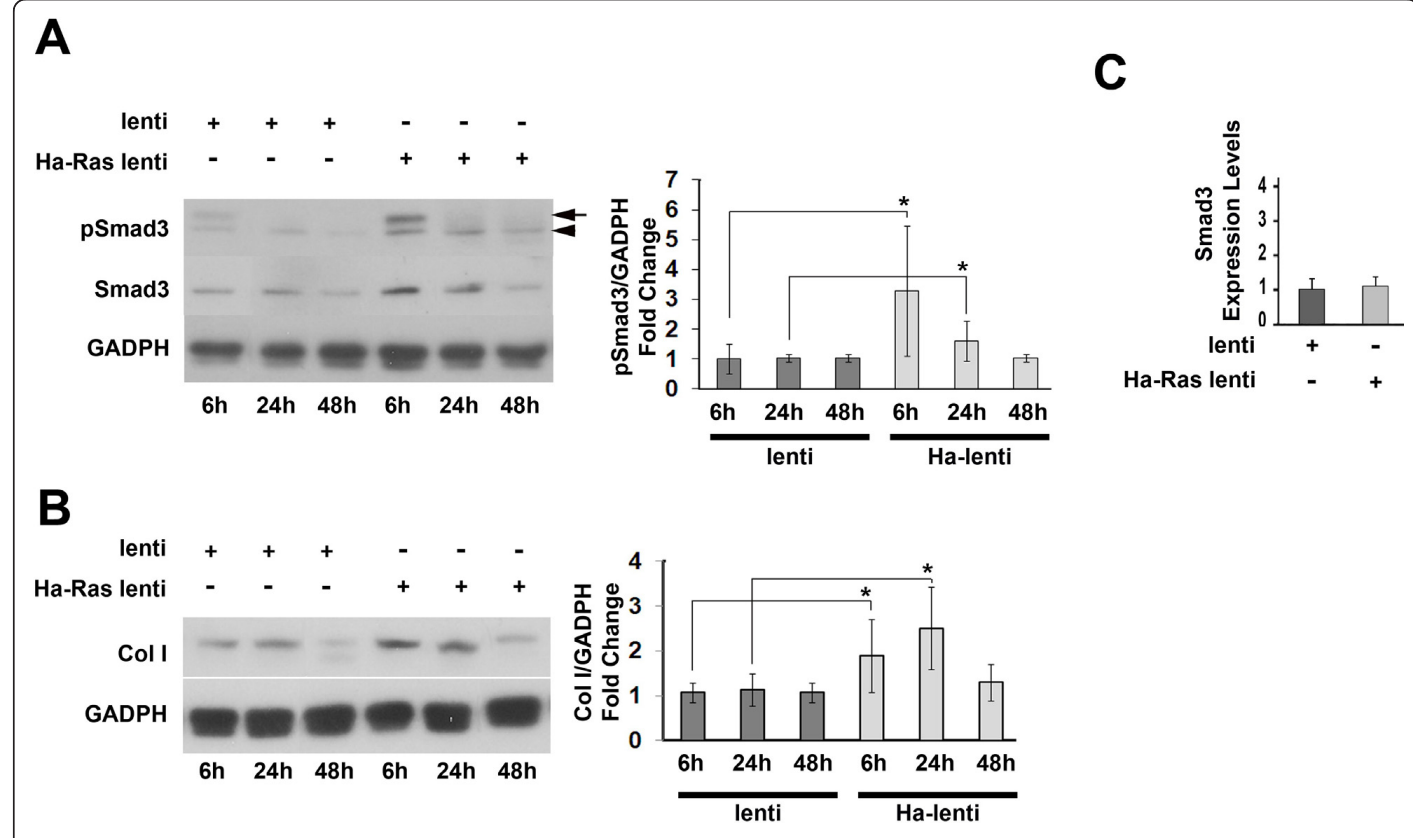
**pSmad3 and collagen type I kinetics in Ha-Ras-infected mouse dermal fibroblast (mDF)**. (A) pSmad3 and Smad3 and (B) collagen I immunoblots (*n *= 3 per each sample) of protein extracts prepared at the indicated time points after infection of mDF cultures with Ha-Ras or control lentiviruses with bar graphs on the side summarizing the ratios of pSmad3 or collagen I relative to the loading control glyceraldehyde 3-phosphate dehydrogenase (GADPH) in the various experimental samples; asterisks indicate statistically significant differences (*P *< 0.05). (C) Real-time quantitative polymerase chain reaction (qPCR) estimates of Smad3 transcript levels in mDF cultures infected with Ha-Ras or control lentiviruses. In the first panel, the arrow and arrowhead respectively point to pSmad3 and an unidentified cross-reacting receptor-activated Smad (R-Smad).

The findings of the transient cell transfection experiments were confirmed in quiescent mDFs that were stably infected with a lentivirus construct expressing Ha-Ras. Specifically, these analyses showed that Ha-Ras expressing mDF display augmented Smad3 phosphorylation and Smad3-reporter plasmid activity (Figure 2A) and collagen I production and COL1A2 promoter transcription (Figure 2B and 2F), as well as more cells expressing contraction-competent actin myofibres compared to mDF infected with control lentivirus (Figure 2C). An additional outcome of Ha-Ras overexpression in mDF cells included the rapid activation of ERK1/2 signalling (Figure 2A), which is part of the self-propagating loop of ROS production, Ha-Ras stabilization and collagen I accumulation in SSc [15,16] and also of JNK and p38 MAPK (data not shown). As a result of its participation in SSc pathogenesis [15,16], ERK1/2 signalling in Ha-Ras over-expressing mDF cells was blunted with the MEK inhibitor PD98059. However, ERK1/2 inhibition had no effect on Ha-Ras-induced stimulation of R-Smad signalling, COL1A2 promoter transcription or collagen I accumulation (Figure 2D-F). Time-point analyses further revealed that R-Smad stimulation is transient and peaks 6 h after mDF infection, whereas collagen I protein levels remain elevated up to 24 h (Figure 3A and 3B). Interestingly, Ha-Ras-expressing cells also exhibited high protein levels of non-phosphorylated Smad3 at 6 h and 24 h post-infection, which were not, however, associated with increased levels of transcripts coding for Smad3 (Figure 3C). We interpreted these last results to suggest that greater amounts of available Smad3 protein may also contribute to heightened pSmad3.

### Proto-oncogene Ha-Ras induces collagen up-regulation independently of TGFβ

**Figure 4:**
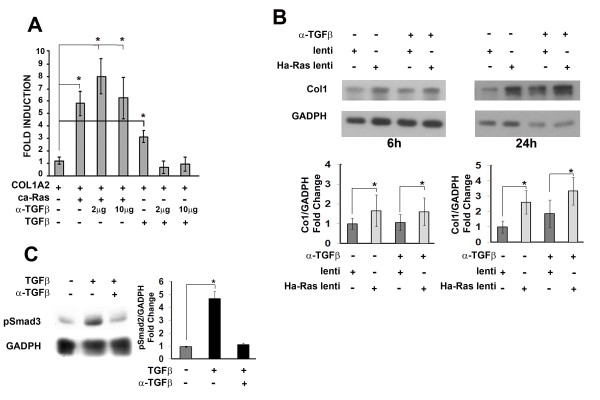
**Ha-Ras up-regulates collagen I independently of autocrine transforming growth factor-β (TGFβ)**. (A) Luciferase activity (expressed as fold induction over control sample) of the human pro-α2 (I) collagen gene (COL1A2) promoter co-transfected with the ca-Ras expressing plasmid in quiescent human dermal fibroblast (hDF) cultured in the presence or absence of TGFβ neutralizing antibody (2 and 10 μg/mL); cells stimulated with recombinant TGFβ1 (2 ng/mL) served as a positive control. Luciferase values represent the average of three independent transfections each performed in duplicate and error bars signify ± standard deviation and asterisks indicate statistically significant differences (*P *< 0.05). (B) Collagen I immunoblots of protein extracts (*n *= 3 per each sample) from mouse dermal fibroblast (mDF) cultures infected with Ha-Ras or control lentiviruses and cultured in the presence or absence of TGFβ neutralizing antibody (10 μg/mL) for the indicated times. (C) pSmad3 immunoblots of protein extracts (*n *= 3 per each sample) from mDF cultures stimulated with recombinant TGFβ1 (2 ng/mL) in the presence or absence of neutralizing TGFβ antibodies (10 μg/mL). In the last two panels, bar graphs summarize the ratio of pSmad3 or collagen I relative to the loading control glyceraldehyde 3-phosphate dehydrogenase (GADPH) in the various experimental samples; asterisks indicate statistically significant differences (*P *< 0.05).

**Figure 5:**
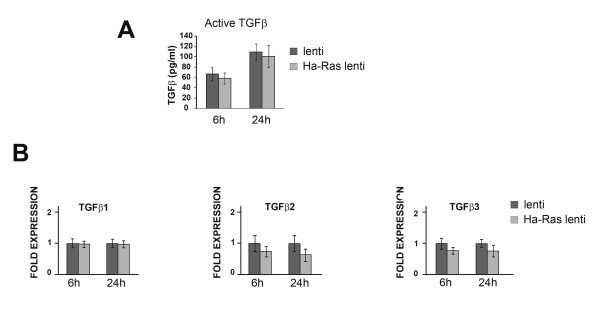
**Normal transforming growth factor-β (TGFβ) levels and activity in Ha-Ras infected mouse dermal fibroblasts (mDFs)**. (A) Cell-based bioassays estimating the amount of active TGFβ in mDF cultures infected with Ha-Ras or control lentiviruses. (B) Real-time quantitative polymerase chain reaction (qPCR) assessment (*n *= 3) of steady-state levels of TGFβ messenger RNAs in mDF cultures infected with Ha-Ras or control lentiviruses. Each bar graph represents three independent samples analysed in duplicate and error bars signify ± standard deviation.

Several reports have shown that Ha-Ras promotes autocrine TGFβ signalling [6,7,20]. The possible contribution of Ha-Ras to TGFβ production and, in turn, collagen I synthesis was therefore investigated in our cell culture systems. To this end, a pan-TGFβ antibody was employed in two complementary experiments which documented the inability of TGFβ antagonism to normalize COL1A2 promoter activity in quiescent hDF cultures transiently co-transfected with the ca-Ras expressing plasmid and in mDF cells stably infected with the Ha-Ras lentivirus (Figure 4A and 4B). These findings excluded the potential involvement of TGFβ neo-synthesis in Ha-Ras up-regulation of collagen I expression. Next, we evaluated whether Ha-Ras overexpression may promote improper activation of latent TGFβ complexes as opposed to TGFβ neo-synthesis. A bioassay, however, revealed comparable levels of active TGFβ in control and Ha-Ras over-expressing mDF cells (Figure 5A). Consistent with this and the above findings, qPCR analyses showed similar levels of TGFβ transcripts in control and Ha-Ras over-expressing mDF cells (Figure 5B). Collectively, the data suggested that increase of Ha-Ras protein levels in dermal fibroblasts promotes an immediate fibrotic response through the canonical R-Smad pathway without establishing an autocrine TGFβ loop.

## Discussion

Accumulation of myofibroblasts and disorganized ECM are the hallmarks of tissue fibrosis. TGFβ is a potent inducer of ECM synthesis and myofibroblasts contraction and a key mediator of wound healing and fibrotic responses [2]. However, TGFβ pleiotropy has largely limited therapeutical intervention in fibrotic diseases, thus stimulating an increased interest in the identification of pro-fibrotic pathways that operate downstream, upstream or in parallel with TGFβ signalling [2]. Data presented here implicate Ha-Ras stabilization in the early onset of fibrosis through TGFβ-independent stimulation of R-Smad signalling.

Previous reports that Ha-Ras proto-oncogene intersects with TGFβ signalling events in several fibrotic conditions [8,9], together with recent evidence that scleroderma auto-antibodies stabilize Ha-Ras levels through ROS action [15,16], led us to hypothesize that increased Ha-Ras activity may influence TGFβ signalling during the early phase of the pro-fibrotic response. Through Ha-Ras overexpression in quiescent mDF, we have demonstrated that the proto-oncogene directly up-regulates collagen production through TGFβ-independent activation of the canonical R-Smad pathway.

Three independent lines of evidence support our conclusion. First, forced expression of DN-Smad3 and mutations in the TbRE of the COL1A2 promoter significantly reduced Ha-Ras-dependent up-regulation of the reporter plasmid. Second, lentiviral overexpression of Ha-Ras rapidly increased Smad3 signalling and R-Smad-dependent reporter activity. Third, mDF pre-treatment with neutralizing pan-TGFβ antibody, or with a MEK inhibitor, showed no appreciable effects on collagen accumulation and Ha-Ras-induced R-Smad activation. These results are in agreement with previous reports indicating that ligand-independent R-Smad signalling is increased in scleroderma cells and that Ha-Ras-dependent MAPK stimulation is not required for R-Smad activation [10,21]. In contrast to the reported participation of ERK1/2 signalling in perpetuating ROS and Ha-Ras stimulation of collagen I production in SSc fibroblasts [15,16], ERK1/2 signalling is not required for collagen I up-regulation in Ha-Ras over-expressing cells. This apparent discrepancy suggests that constitutively high levels of Ha-Ras in our cell culture system do not require the postulated feed-forward loop of ERK1/2 signalling [15,16]; implicitly, our conclusion suggests that the amount of Ha-Ras is a limiting factor during the early phase of SSc fibrosis.

Protein turnover is another modulator of R-Smad3 activity in addition to protein phosphorylation [4]. The finding that Ha-Ras overexpression in mDF is associated with increased Smad3 protein levels and normal amounts of *Smad3 *transcripts strongly suggests decreased protein degradation. Recently, the protein kinase GSK3β has been shown to control Smad3 ubiquitination and degradation [22]. GSK3β activity is negatively regulated by Ras family members and fibroblast-specific deletion of GSK3β in mice results in accelerated wound closure, increased fibrogenesis and excessive scarring [23,24]. Altogether, these reports and our results are at least consistent with the notion that augmented Smad stability in mDF overexpressing Ha-Ras accounts in part for the ligand-independent increase of canonical TGFβ signalling and collagen production. A similar situation has been described for SSc fibroblasts in which higher than normal levels of Smad3 and Ha-Ras proteins are both associated with increased collagen accumulation [16,21].

In contrast with our observations, others have reported that oncogenic Ha-Ras (*V12*-Ha-Ras) dictates the response of cancer cells to TGFβ by decreasing Smad3 stability, thus suggesting a negative role of Ha-Ras in modulating R-Smad signalling [25]. Moreover, Ha-Ras has been shown to inhibit R-Smad activity in epithelial cells and to down-regulate collagen expression in proliferating fibroblasts [26,27]. We believe that these apparent discrepancies probably reflect the multiple roles that Ha-Ras plays in integrating the contextual responses of cells to TGFβ signalling. Our results contribute to the ongoing efforts to dissect the complex network of signalling events that drive the onset and progression of tissue fibrosis.

## Conclusions

Our results provide a mechanistic insight into the role of Ha-Ras stabilization in driving collagen I overproduction during the early phase of dermal fibrosis in SSc by showing a direct involvement of the proto-oncogene in stimulating R-Smad signalling independently of TGFβ neo-synthesis or activation and of ERK1/2 signalling. This conclusion is based on *in vitro *evidence that correlated Ha-Ras-induced activation of R-Smad directly to the stimulation of COL1A2 promoter reporter plasmid and with the elevation of endogenous collagen I protein. Together, our findings extend and refine recent reports that implicated circulating PDFR auto-antibody in the triggering of the ROS-mediated stabilization of Ha-Ras activity in SSc [15,16]. As such, they contribute to a better understanding of the early signalling events and potential therapeutic opportunities in this acquired disabling disorder of the connective tissue.

## Abbreviations

αSMA: α-smooth muscle actin; ca-Ras and DN-Ras: constitutively active and dominant-negative Ha-Ras, respectively; COL1A2: human pro-α2 (I) collagen gene; DMEM: Dulbecco's modified Eagle medium; DN-Smad3: dominant-negative Smad3; ECM: extracellular matrix; hDF and mDF: human and mouse dermal fibroblasts, respectively; FBS: fetal bovine serum; MAPK: mitogen-activated protein kinase; PDGFR: platelet-derived growth factor receptor; p-Smad, R-Smad: phosphorylated and receptor-activated Smad, respectively; qPCR: real-time quantitative polymerase chain reaction; ROS: reactive oxygen species; SSc: scleroderma; TbRE: TGFβ-responsive element; TGFβ: transforming growth factor-β.

## Competing interests

The authors declare that they have no competing interests.

## Authors' contributions

SS and JO carried out all the experiments and data analyses. LG provided the lentiviral vectors and relevant protocols, in addition to supervising the lentivirus infection experiments. GM performed TGFβ assays with scleroderma auto-antibodies that were not included in the present manuscript. AG and FR designed the experiments and interpreted the data. SS and FR wrote the final manuscript. All authors have read and approved the final manuscript.
